# Does an Adductor Canal Block Influence Patient-Reported Outcomes at One Year Following Total Knee Arthroplasty?

**DOI:** 10.7759/cureus.41123

**Published:** 2023-06-29

**Authors:** Edward D McKee, Nick D Clement

**Affiliations:** 1 General Medicine, Victoria Hospital, Kirkcaldy, GBR; 2 Orthopaedics, Royal Infirmary of Edinburgh, Edinburgh, GBR

**Keywords:** one year outcomes, patient-reported outcome measures, total knee arthroplasty, local anaesthetic wound infiltration, adductor canal block

## Abstract

Introduction

Total knee arthroplasty (TKA) for osteoarthritis is performed to improve knee function and quality of life. Adductor canal block (ACB) (with posterior capsule local anaesthetic) and periarticular local anaesthetic infiltration (LA) alone are common methods used for post-operative pain control following TKA. The primary aim of this study was to investigate the influence of ACB compared to LA alone on knee function at one year in patients undergoing primary TKA. The secondary aims were to investigate the influence on health-related quality of life (HRQoL), patient satisfaction at one year, and length of stay (LOS) in hospital following TKA.

Methods

During a three-year period, 1396 patients who underwent TKA at the study centre completed pre-operative and one-year post-operative questionnaires. Data collected included patient demographics, co-morbidities, LOS, Oxford Knee Score (OKS), EuroQol 5-Dimension (EQ-5D) and satisfaction scores. Patients who had a spinal anaesthetic (n=1096) with either ACB (with posterior capsule local anaesthetic) (n=224) and LA infiltration alone (n=872) were compared.

Results

There was a significant improvement in pre-operative to one-year post-operative knee function as measured by OKS overall (15.1, p<0.001), however there was no significant difference between ACB and LA groups (p=0.37). There was no significant difference in change (p=0.43) or one-year post-operative (p=0.70) HRQoL, as measured by EQ-5D. There was also no significant difference in one-year patient satisfaction between groups (p=0.57). There was no significant difference in median LOS between groups (four days with ACB, four days with LA), although patients younger than 55 years undergoing ACB had a statistically significant median of one day reduced LOS compared with the LA group (p=0.01).

Conclusions

ACB when compared with LA alone was not associated with a difference in change in knee function, HRQoL, or patient satisfaction at one year following TKA. There was also no difference between groups in length of stay post-operatively. However, ACB was associated with a shorter length of stay in patients younger than 55, and this may be a group that would benefit from this intervention.

## Introduction

The outcome of a total knee arthroplasty (TKA) can be negatively influenced by many different factors, such as technical and medical complications, co-morbid conditions, higher pre-operative pain level, and psychological determinants [1-4]. Post-operative pain can also significantly influence outcome [5]. Unfortunately, 60% of patients experience severe pain in the initial post-operative period [6], preventing early physiotherapy and mobilisation. The adoption of early movement and strengthening exercises is the most significant factor in post-operative knee rehabilitation, decreasing both length of stay (LOS) in hospital and incidence of post-operative complications [7,8]. In addition, post-operative pain is associated with reduced knee range of motion and increased risk of arthrofibrosis, adhesions, capsular contracture and muscle atrophy [9,10]. These can have long-term negative effects on the function of the operated knee.

Opioids, commonly used to control post-operative pain, can have many adverse effects including nausea, pruritus, respiratory depression and sedation [7]. Tolerance and physical dependence can occur, with a significant risk of persistent use. Adjuvant analgesic modalities such as peripheral nerve block (PNB) and local anaesthetic infiltration (LA) are now frequently used in clinical practice as opioid-sparing post-operative pain management techniques [11]. The use of PNB with regional anaesthesia has been shown in multiple studies to reduce post-operative pain, reduce length of stay, and increase mobilisation of those without PNB (using oral analgesia alone as an adjunct to regional anaesthesia) [12]. Adductor canal block (ACB) is a form of PNB. Requiring an experienced anaesthetist, an ultrasound transducer is used to guide LA injection anterolateral to the femoral artery and deep to the posterior fascia of the sartorius muscle [13,14]. ACB produces sensory blockade in the saphenous nerve, posterior branch of obturator nerve, and vastus medialis nerve distribution [15]. ACB minimally affects quadriceps strength if performed correctly, allowing earlier post-operative rehabilitation and reduced risk of falls than PNB [14]. In our study centre, when undergoing ACB, the surgeon also infiltrates local anaesthetic into the posterior capsule to provide analgesia to the posterior aspect of the knee.

Periarticular LA infiltration was first reported in 2008, whereby an analgesic combination (often local anaesthetic, a non-steroidal anti-inflammatory drug, and adrenaline) is injected into the surgical site intra-operatively, with a further injection 15-20 hours post-operatively through intra-articular catheter [16]. LA has minimal effect on motor function post-operatively and has been shown in meta-analyses to reduce post-operative opioid requirements, nausea and vomiting, and LOS [17,18]. LA infiltration is easier than ACB to perform in clinical practice and may only increase intra-operative time by around 9 minutes [19].

ACB has been compared in literature with LA infiltration on short-term pain outcomes following TKA using data such as post-operative opioid consumption and visual analogue pain scales [13,20]. However, there have been few studies comparing ACB and LA infiltration on longer-term functional outcomes post-TKA. The primary aim of this study was to investigate the influence of ACB in comparison with LA alone on knee function at one year in patients with primary TKA. Our secondary objectives were to investigate the influence on health-related quality of life (HRQoL), patient satisfaction at one year and LOS in hospital following TKA.

## Materials and methods

Patients were identified retrospectively from electronic theatre records covering 1396 patients undergoing a primary TKA in Edinburgh Royal Infirmary from 2014-2016. These patients had completed a pre-operative and one-year post-operative questionnaire including demographics and co-morbidities. The Oxford Knee Score (OKS) (zero = worst function, 48 = best function), EuroQol 5-Dimension (EQ-5D) score (one = full health, zero = death, negative number = state worse than death) and satisfaction scores were recorded both pre-operatively and one year post-operatively. The National Health Service (NHS) uses the OKS as the standard patient-reported outcome measure (PROM) to determine knee function, and the collection of PROMs is now mandatory for patients undergoing knee arthroplasty. Both the OKS and EQ-5D scores are reproducible, valid and clinically sensitive. To determine satisfaction, patients completed a Likert satisfaction score one year after surgery (one = very satisfied, two = satisfied, three = neither, four = dissatisfied, five = very dissatisfied). The type of anaesthesia with post-operative pain management technique given to each patient was categorised as spinal anaesthesia with local anaesthetic infiltration (LA), spinal anaesthesia with adductor canal block (with concurrent posterior capsule LA) (ACB), and general anaesthesia. For our study, the LOS for each patient was found by searching for the date of surgery and discharge for each patient using their unique identifier on the patient record system, TrakCare, and added to the database for analysis. 

Included in the analysis were patients who underwent a primary TKA for primary osteoarthritis (OA) under spinal anaesthesia. Exclusion criteria covered patients who had a TKA for a condition other than OA, bilateral TKA, patients with incomplete data, and those who had undergone TKA under general anaesthesia. If a patient had a TKA on both knees during the study period, only the data from the first TKA was included. All TKAs were performed by one of 18 consultants, and all patients underwent a standard rehabilitation programme. Ethical approval was granted by the South East Scotland Research Ethics Service. 

Data were analysed using Statistical Package for Social Sciences v29.0 (IBM Corporation, Armonk, NY, USA). The types of post-operative pain management were compared on functional outcome, HRQoL, patient satisfaction (all at one year), and LOS post-operation. Analysis was performed on the ACB and LA groups using independent sample t-tests (with chi-square analyses for categorical data). Tests for normality (histogram, Q-Q plot, skewness and kurtosis) were performed and the Mann-Whitney U test was used to compare non-parametric linear variables between groups. Regression analysis was used to assess for the independent association of ACB when compared to LA alone on change in the OKS and EQ-5D (linear), and patient satisfaction (logistic) at one year. A p-value of <0.05 was used to show statistical significance of results throughout. 

## Results

There were 300 patients (21.5%) excluded from the analysis as they did not meet the inclusion criteria. The study group consisted of 504 males (46.0%) and 592 (54.0%) females. The mean age of male patients was 69.8 years (45 to 91) and the mean age of female patients was 70.7 years (47 to 91), which was not a significant difference (p=0.10, independent t-test) (Table 1). Eight hundred seventy-two (79.6%) of patients who met the inclusion criteria underwent the operation under spinal anaesthesia with LA alone, while 224 (20.4%) underwent the operation under spinal anaesthesia with ACB (Table 1). There was no significant difference in sex (p=0.88; chi-squared), age (mean difference 1.2; p=0.07, t-test), pre-operative OKS (mean difference 0.6; p=0.32, t-test) or pre-operative EQ-5D (mean difference 0.03; p=0.24, t-test) between those who had ACB and LA alone (Table 1). However, there was a significant difference in the American Society of Anesthesiologists (ASA) grade between the groups, with the ACB group having a greater number of ASA grade 2 patients compared to the LA group (p=0.02, chi-squared) (Table 1). 

**Table 1:** Demographics of the post-operative pain management groups ACB: Adductor Canal Block, LA: Local Anaesthetic Infiltration, ASA: American Society of Anesthesiologists, SD: Standard Deviation, CI: Confidence Interval *independent t-test **chi-square

		ACB (n = 224)	LA (n = 872)	Difference (95% CI)	p-value (*)
Age, years (SD)		69.3 (8.9)	70.5 (8.8)	1.2 (-0.08 to 2.5)	0.07
				p-value (**)	
Sex	Male, n (%)	104 (46.4)	400 (45.9)	0.88	
	Female, n (%)	120 (53.6)	472 (54.1)		
ASA	1, n (%)	20 (8.9)	82 (9.4)	0.02**	
	2, n (%)	177 (79.0)	630 (72.2)		
	3, n (%)	25 (11.1)	158 (18.1)		
	4, n (%)	1 (0.4)	0 (0.0)		

The overall mean OKS change from pre-operation to one-year post-operation was 15.1, which was significant (p<0.001, paired t-test) (Table 2). The mean OKS difference from pre-operation to one-year post-operation was 15.6 in the ACB group and 15.0 in the LA group, however the difference between groups was not significant (p=0.37, t-test) (Table 2). When adjusting for confounding factors using regression analysis there remained no significant difference between groups for change in OKS at one year (Table 3). Overall, males had a significantly higher pre-operative OKS (mean difference 3.0; p<0.001, t-test) and post-operative OKS (mean difference 1.6; p=0.005, t-test) than females. However, females had a significantly larger mean pre-operative to post-operative OKS improvement than males (mean difference 1.4; p=0.02, t-test). The mean EQ-5D change from pre-operation to one-year post-operation was 0.33 in the ACB group and 0.31 in the LA group, which was not a significant difference (p=0.43, t-test) (Table 2). There was also no significant difference in post-operative EQ-5D between ACB and LA groups (p=0.70, t-test) (Table 2). When adjusting for confounding factors using regression analysis there remained no significant difference between the groups for change in the EQ-5D at one year (Table 3). Both groups, however, showed a significant improvement in HRQoL pre- to post-operation (p<0.001, paired t-test) (Table 2). Females had a significantly greater mean improvement in their EQ-5D when compared to males (mean difference 0.06; p=0.004, t-test). 

**Table 2:** Oxford Knee Score and Euro-Qol 5-Dimension scores pre- to post-operatively within and between pain management groups PROM: Patient Reported Outcome Measure, SD: Standard Deviation, ACB: Adductor Canal Block, LA: Local Anaesthetic Infiltration, EQ-5D: Euro-Qol 5-Dimension, OKS: Oxford Knee Score, CI: Confidence Interval *independent t-test  **paired t-test

PROM	ACB (n = 224)	LA (n = 872)	Difference (95% CI)	p-value (*)
OKS				
Pre-operative (SD)	20.8 (7.6)	21.4 (7.8)	0.6 (-0.6 to 1.7)	0.32
Post-operative (SD)	36.4 (9.4)	36.3 (9.4)	0.1 (-1.4 to 1.3)	0.94
Pre- to Post- Operative Difference (SD)	15.6 (9.8)	15.0 (9.3)	0.6 (-2.0 to 0.7)	0.37
p-value (**)	p<0.001**	p<0.001**		
EQ-5D				
Pre-operative (SD)	0.42 (0.31)	0.45 (0.30)	0.03 (-0.02 to 0.07)	0.24
Post-operative (SD)	0.75 (0.25)	0.76 (0.25)	0.01 (-0.03 to 0.04)	0.70
Pre- to Post-operative Difference (SD)	0.33 (0.34)	0.31 (0.33)	0.02 (-0.07 to 0.03)	0.43
p-value (**)	p<0.001**	p<0.001**		

**Table 3:** Multivariable regression analysis adjusting for pre-operative confounders (age, sex, ASA grade, socioeconomic status, preoperative OKS and EQ-5D) of pain management technique on post-operative outcomes PROM: Patient Reported Outcome Measure, LA: Local Anaesthetic Infiltration, ACB: Adductor Canal Block, OKS: Oxford Knee Score, EQ-5D: Euro-Qol 5-Dimension, CI: Confidence Interval, ASA: American Society of Anesthesiologists

PROM and Timepoint	R^2 ^	Group	Mean Difference	95% CI		p-value
				Lower	Upper	
OKS	0.199	LA	Reference			
		ACB	0.3	-1.0	1.5	0.704
EQ-5D	0.506	LA	Reference			
		ACB	0.002	-0.034	0.037	0.932
			Odds Ratio			
Satisfaction	0.030	LA	Reference			
		ACB	1.06	0.72	1.57	0.765

**Figure 1:**
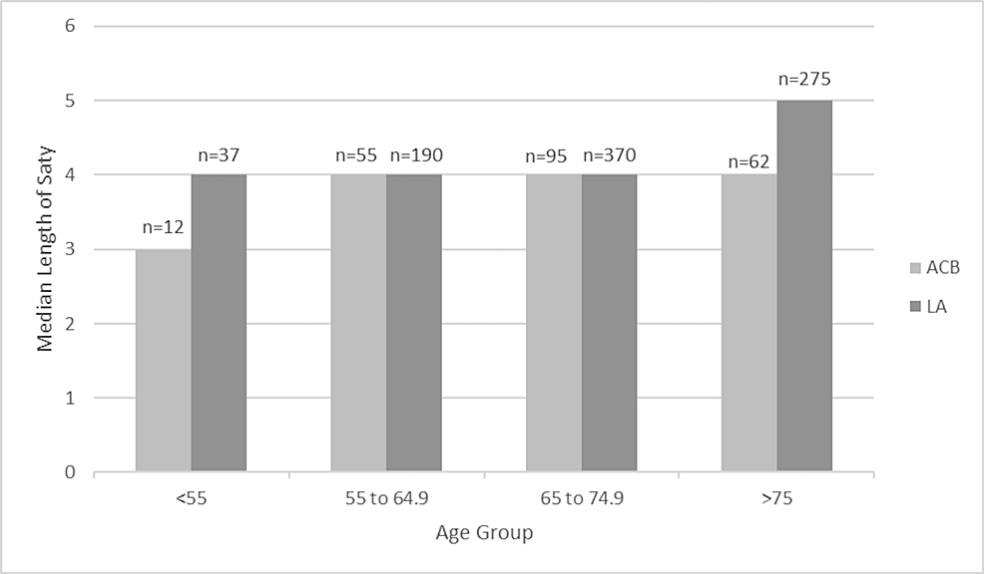
The median length of stay for each age group, split by pain management technique LA: Local Anaesthetic Infiltration, ACB: Adductor Canal Block, n: number of patients

The median LOS of all patients was four days. The median LOS was four days in the ACB group and four days in the LA group (p=0.48, Mann-Whitney), and therefore was not significantly different between groups. There was a significant correlation between increasing age and increasing length of hospital stay (r=0.24, p<0.001, Spearman’s). For patients less than 55 years old, those having an ACB had a median of one day less in hospital post-operatively compared to the LA group (median three versus four days, p=0.01; Mann-Whitney) (Figure 1). There was no significant difference in ASA grade between groups in this age bracket (p=0.56, chi-squared). The median LOS in 2014 overall was four days and remained at four days in 2016. There was no difference in median LOS between females and males (both four days).

Twenty patients (1.8%) did not complete post-operative satisfaction scores. Eight hundred ninety-one patients (83%) were satisfied with their operation. One hundred fifteen (11%) were neutral and 70 (7%) were dissatisfied. No association was found between post-operation pain management and satisfaction (p=0.57, chi-squared). When adjusting for confounding factors using logistic regression analysis there remained no significant difference between the groups for satisfaction at one year. Satisfaction with TKA at one year was equal in both groups (83%), and was overall associated with both post-operative OKS and OKS change (p<0.001). 

## Discussion

ACB when compared with LA alone was not associated with a difference in knee-specific function, HRQoL, or patient satisfaction at one year following TKA. There was no difference in overall LOS between the groups, however patients younger than 55 years receiving an ACB had a one-day shorter LOS compared to those receiving LA alone. 

It has been shown total morphine consumption at 24 and 48 hours post-TKA is lower in patients undergoing ACB than LA [13]. This is important, as better pain control has been shown to be significantly associated with improved functional outcomes at three months [5]. ACB has also been shown to have a significantly better Timed Up and Go Test (TUG) score at post-operative day 3 than LA, although quadriceps strength in both groups was similar [13]. A better early TUG score and reduced pain could allow patients who have had ACB to engage with physiotherapy earlier and therefore aid their rehabilitation [8]. However, other factors may be more important in determining functional ability at one year (such as continued engagement with physiotherapy, co-morbidities, and initial indication for surgery) and therefore obscure any measurable benefit of ACB over LA alone. 

There was no significant change in HRQoL as measured by EQ-5D between groups, although both groups had a significant improvement from pre-operation to one-year post-operation. It has been shown in one study that HRQoL as measured by EQ-5D improved post-operatively when measured at 10 months and further substantially improved when re-measured at 11-22 months [21]. Therefore, follow-up at one year may not be sufficiently long enough post-operation to elicit the full positive impact on HRQoL. Alternatively, as with functional knee outcome, the follow-up questionnaire being at one year may have meant any effect on HRQoL attributable to post-operative pain-management technique may be masked by other factors. Post-operative EQ-5D has been shown to be predicted by age, sex, education, obesity, socioeconomic status, anxiety and depression [22]. A regression analysis was completed including available confounding factors in our study (sex, socioeconomic group, ASA grade, pre-operative functional scores), and there remained no significant difference in EQ-5D change between groups. 

There was no significant difference between groups in LOS. This corroborates a meta-analysis of randomised control trials comparing LOS with ACB or LA [23]. Both groups should be able to complete early physiotherapy and ambulation, unlike with the use of PNB, which causes temporary motor block to the quadriceps. Therefore, factors other than post-operative motor function of the leg may be more influential on the LOS in our study, for example post-operative pain, which is associated with LOS [23], and peri-operative complications. Unfortunately, post-operative pain scores and peri-operative complications were not recorded in our database; this has been noted as a limitation of our study. The cost per day as an inpatient on an orthopaedic ward following TKA has been calculated in a previous study at £487 [24]. Patients aged younger than 55 years had a statistically significant one-day reduction in median LOS when undergoing an ACB as compared to LA alone. Previous studies have not included data on LOS by age group when comparing ACB and LA, and it is unclear why in this study this demographic has benefitted. Further research with a higher volume of younger patients is necessary, given the importance of shorter LOS for both reducing hospital-acquired complications and cost of inpatient stay. 

The cost-effectiveness of ACB may vary between hospitals due to experience and availability of anaesthetists [12]. It has recently been shown that intra-articular surgeon-administered ACB is not inferior to ACB administered by an anaesthetist on patient satisfaction, opioid use, or PROMs [25]. However, theatre time is the costliest component of ACB (£20.50 per minute) [24], and extending this may negate any savings in cost of pre-theatre anaesthetist time. The cost of LA was not calculated in this study, however LA infiltration in addition to a standard anaesthetic regime has been shown to save £77 per patient over anaesthetic regime alone [26]. The main cost of LA infiltration intra-operatively is the cost of theatre time, and it has been shown to take on average 9 minutes [19]. 

Our study found that 83% of patients were satisfied one year post-operatively, similar to other studies [27]. Satisfaction may increase further with longer follow-up - it has been shown in one study that the percentage of patients either ‘enthusiastic’ or ‘satisfied’ increased from 88% at three years to 92% at 10 years [28]. There was no significant difference in satisfaction between groups in our study. This agrees with other studies that have shown both LA and ACB lead to similarly high patient satisfaction [13]. Satisfaction has also been shown previously to correlate with functional score [27]. Our study confirmed this, showing that satisfaction is significantly associated with post-operative OKS and pre- to post-operative OKS change. 

There were limitations to our study, given its retrospective nature. Peri-operative complications, failures of TKA and post-operative pain scores were not collected. Data was not collected on failed ACBs leading to conversion to LA, which would increase costs of the operation substantially. Every TKA was carried out by a consultant surgeon, however difference in experience and technique may have still influenced results. The effects of the spinal anaesthesia itself persist into the post-operative period and make it more difficult to determine relative efficacy of ACB and LA. Bupivacaine’s effects persist 1-1.5 hours after surgery, and ropivacaine 0.5% may last 24 hours, although anaesthetists highly trained in ultrasound-guided regional anaesthesia can avoid this [20]. Furthermore, the exact combination and volume of local anaesthetics infiltrated were not standardised; this could make results more difficult to replicate. Findings of the study should therefore be interpreted within the context of the limitations above. A prospective randomised control trial comparing the two groups, including data on complications and extended follow-up could avoid some of the limitations and corroborate the findings of a retrospective study. 

## Conclusions

It is vitally important to identify and address factors which may improve patient outcomes following TKA. The efficacy of post-operative pain management is known to affect functional outcomes, and two common methods of this were compared in this study. A peri-operative ACB was not associated with significantly different one-year functional outcomes when compared to LA infiltration alone following TKA. There was no significant difference in patient satisfaction between groups. There was no difference between groups in length of stay post-operatively, however patients younger than 55 years undergoing an ACB had a one-day shorter LOS in hospital. Further research into the reasons for the reduced LOS in this age group could be important due to the benefits of minimising post-operative stay. 
